# Intracellular sphingolipid sorting drives membrane phase separation in the yeast vacuole

**DOI:** 10.1016/j.jbc.2023.105496

**Published:** 2023-11-25

**Authors:** Hyesoo Kim, Itay Budin

**Affiliations:** Department of Chemistry and Biochemistry, University of California San Diego, La Jolla, California, USA

**Keywords:** lipid rafts, lysosome, sphingolipids, ergosterol, microautophagy

## Abstract

The yeast vacuole membrane can phase separate into ordered and disordered domains, a phenomenon that is required for micro-lipophagy under nutrient limitation. Despite its importance as a biophysical model and physiological significance, it is not yet resolved if specific lipidome changes drive vacuole phase separation. Here we report that the metabolism of sphingolipids (SLs) and their sorting into the vacuole membrane can control this process. We first developed a vacuole isolation method to identify lipidome changes during the onset of phase separation in early stationary stage cells. We found that early stationary stage vacuoles are defined by an increased abundance of putative raft components, including 40% higher ergosterol content and a nearly 3-fold enrichment in complex SLs (CSLs). These changes were not found in the corresponding whole cell lipidomes, indicating that lipid sorting is associated with domain formation. Several facets of SL composition—headgroup stoichiometry, longer chain lengths, and increased hydroxylations—were also markers of phase-separated vacuole lipidomes. To test SL function in vacuole phase separation, we carried out a systematic genetic dissection of their biosynthetic pathway. The abundance of CSLs controlled the extent of domain formation and associated micro-lipophagy processes, while their headgroup composition altered domain morphology. These results suggest that lipid trafficking can drive membrane phase separation *in vivo* and identify SLs as key mediators of this process in yeast.

The ability of lipid-lipid interactions to drive heterogeneity within continuous bilayers has been proposed as a mechanism for organization in cell membranes. The lipid raft hypothesis postulates that biological membranes can phase separate into coexisting liquid-ordered (Lo) and liquid-disordered (Ld) domains based on lipid composition and temperatures (1). Sterols, such as cholesterol or ergosterol, and lipids with saturated acyl chains, including sphingolipids (SLs), are required to form Lo domains and can thus be referred to as raft components. In contrast, Ld domains are enriched in unsaturated glycerophospholipids (GPLs) (2). Lipid rafts were first proposed based on the co-segregation of lipids and proteins into detergent-resistant fractions of apical epithelial membranes (3) and later characterized extensively in synthetic giant unilamellar vesicles (GUVs) composed of ternary mixtures of lipids (4). However, large Lo membrane domains are not readily observable in mammalian cells (5) and current models for lipid rafts focus on small (<100 nm), dynamic assemblies only accessible by superresolution imaging approaches (6, 7, 8).

While initial work on membrane domains focused on the mammalian plasma membrane (PM), the vacuole of budding yeast (*Saccharomyces cerevisiae*) has become a surprising and robust model for micron-scale membrane phase separation. Vacuoles are digestive organelles similar to mammalian lysosomes (9) and their membrane organization has been implicated in microautophagy-related degradation of energy stores during nutritional starvation (10). Freeze fracture electron microscopy studies first suggested that vacuole membranes can display lateral heterogeneity that is dependent on growth stage (11, 12, 13). More recently, this phenomenon has been explored using fluorescence microscopy. In exponentially growing cells, vacuole membrane-associated proteins expressed as fluorescent protein fusions are evenly distributed across the surface, but in stationary stage segregate into discrete patterns of domains and surrounding areas (14). Vacuole domains, labeled with Lo proteins such as the BAR-domain containing protein Ivy1, are often polygonal in shape and are stained with the sterol-binding dye filipin. In contrast, the surrounding areas are labeled with Ld proteins, such as the vacuolar ATPase Vph1 or alkaline phosphatase Pho8 (15), and are stained with lipophilic dyes that localize to Ld domains in GUVs, like FAST DiI. Vacuole domains dissolve above a characteristic melting temperature and this process is reversible (16), similar to domain formation in phase-separated GUVs. In cells, vacuole phase seperation is dependent on autophagy-related machinery and the resulting Lo domains serve as docking sites for lipid droplet (LD) internalization (17). Once in the vacuole, LDs are digested to release fatty acids that drive energy metabolism via β-oxidation during conditions of glucose deprivation, a process termed micro-lipophagy (18). Cells lacking vacuole domains are defective in micro-lipophagy and show poor survival under nutritional stress, while those lacking autophagy machinery fail to form vacuole domains and degrade LDs (17).

Although biological functions for vacuole domains have been identified, the specific changes in membrane lipid or protein composition that drive their formation have not yet been defined. Sterols, which are predominantly in the form of ergosterol in fungi, are hallmarks of Lo domains and are required for their formation *in vitro* (4). Vacuole domains are lost upon ergosterol extraction by methyl-β-cyclodextrin and fail to form when cells are grown with the ergosterol biosynthesis inhibitor fenpropimorph (14). Reduction in whole cell ergosterol levels through repression of sterol biosynthesis also modulates vacuole domain abundance and size (19). Ergosterol and other lipids could be trafficked to the vacuole membrane via vesicular pathways, such as the vacuole fusion of multivesicular bodies (MVB) (20) or autophagosomes (17). Non-vesicular machinery in the vacuole, such as the Niemann-Pick type C (NPC) homologs Ncr1 and Npc2 (15, 21), could also be relevant for the incorporation of internalized lipids from the vacuole lumen into the surrounding membrane. Loss of Vps4 (14), an AAA-ATPase involved in the genesis of MVBs, essential autophagy genes like Atg8 (17), or the yeast NPC proteins (21) all reduce the frequency of membrane domains, suggesting that lipid trafficking to the vacuole membrane could be involved in its phase separation.

To form Lo domains, sterols must preferentially interact with saturated lipids, which in eukaryotic cells are predominantly SLs. In contrast to metazoans, complex sphingolipids (CSLs) in yeast are anionic and contain one of three headgroups composed of inositol phosphate and mannose. The resulting CSLs—inositol phosphorylceramide (IPC), mannosyl inositol phosphorylceramide (MIPC), and mannosyl diinositol phosphorylceramide (M(IP)_2_C)—are among the most abundant lipids in yeast cells, together comprising 10 to 15 mol % of the lipidome (22). Overall CSL concentration stays constant during yeast growth stages, but the stoichiometry between the three species changes (23). CSLs are formed from a ceramide (either phytoceramides or dihydroceramide) backbone that feature very long chains, with a combined length of up to 46 carbons, that can be additionally hydroxylated (22). During SL metabolism, phytoceramides or dihydroceramides are first synthesized in the Endoplasmic Reticulum (ER), transferred to the Golgi for modification, and then sorted for transport to other compartments (24, 25, 26).

It has been proposed that domain formation in stationary stage or starved cells occurs via a redistribution of raft-forming lipids within the cell, leading to an increase in their abundance under these conditions (19). Directly testing this model has been hindered by the challenges in isolating stationary stage vacuoles, including poor cell wall lysis and growth stage differences in vacuole density. Recently, a method for immunoprecipitation of vesicles derived from yeast organelles has been developed, termed MemPrep (27), and has been applied to the analysis of stationary vs. exponential stage vacuoles sampled at 48 and 8 h of growth, respectively (28). Stationary stage vacuole membranes isolated using the bait vacuole protein Mam3, an Ld marker, showed no increase in ergosterol content, and overall low levels of both ergosterol and SLs. Instead, an increase in the abundance of high melting temperature phosphatidylcholine (PC) species was observed, which also increased in the corresponding whole cell lipidome.

In this study, we test the hypothesis that redistribution of raft-forming lipids into the vacuole drives its membrane to phase separate under glucose restriction. We find that the lipidomes of early stationary stage vacuoles isolated by density centrifugation show a clear enrichment in Lo domain-promoting lipids, especially CSLs that increase several fold in abundance compared to late exponential stage vacuoles. These increases are not found in the corresponding whole cells. We then genetically target the yeast CSL pathway to dissect how lipid composition controls vacuole domain abundance, morphology, and microautophagy of LDs during nutritional stress.

## Results

### Lipidomic analysis of isolated yeast vacuoles under domain-forming conditions

**Figure 1:**
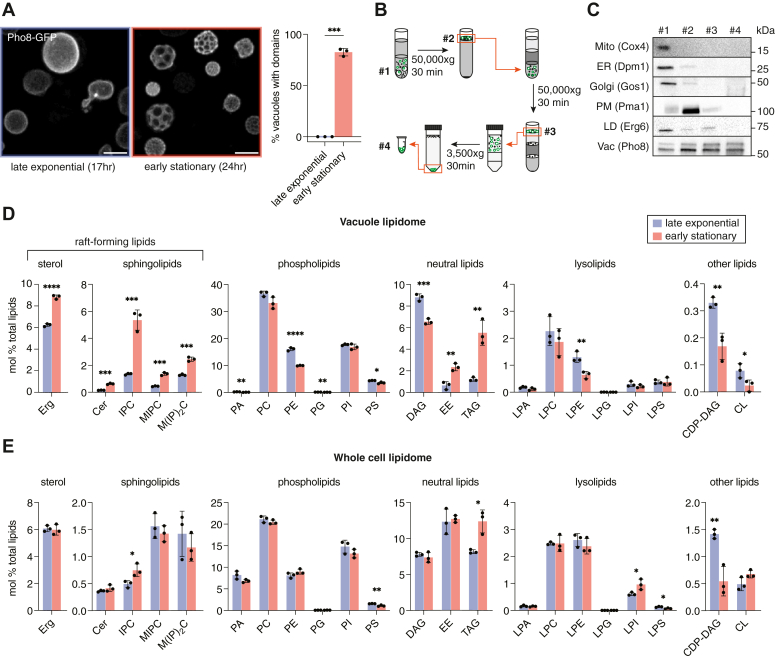
**Lipidomic changes accompanying phase separation in the yeast vacuole**. *A*, vacuole membranes phase separate into discrete domains during a short growth period (7 h) as cells enter the stationary stage. 3D projections shown are from confocal Z-stacks of a field of live cells expressing the vacuole Ld domain marker Pho8-GFP grown under vacuole isolation conditions. Scale bar, 3 μm. The bar graph shows the fraction of cells grown under early stationary stage isolation conditions show phase separation, while none do under the late exponential stage conditions. Error bars represent SD for n = 3 individual cultures; N > 100 cells per replicate. *B*, centrifugation-based isolation of stationary stage vacuoles. Vacuoles were separated from cell lysates via two rounds of density gradient ultracentrifugation, followed by a low speed centrifugation step to remove associated LDs. *C*, western blot analysis of the indicated fractions in the diagram shows how the purification method successively depletes non-vacuole components from stationary stage vacuoles. Quantification of impurities and equivalent data for late exponential vacuoles is shown in Fig. S1. *D*, the abundance, expressed as a mol % of total lipids, for each lipid class in vacuoles isolated from W303a cells in late exponential stage (17 h of growth, *blue*) and early stationary stage (24 h of growth, *red*) in minimal medium. Error bars indicated SD for n = 3 replicate preparations from individually grown cultures. Significance was assessed by unpaired two-tailed *t* test with all comparisons shown; ∗*p* < 0.05; ∗∗*p* < 0.01; ∗∗∗*p* < 0.001. *E*, the corresponding lipidomes from intact whole cells grown identically as for vacuole purification, which do not show increases in raft-forming lipids. CDP-DAG, cytidine diphosphate-diacylglycerol; Cer, ceramide; DAG, diacylglycerol; Erg, ergosterol; LPA, lysophosphatidic acid; LPC, lysophosphatidylcholine; LPE, lysophosphatidylethanolamine; LPG; lysophosphatidylglycerol; LPI, lysophosphatidylinositol; LPS, lysophosphatidylserine; PG, phosphatidylglycerol; PI, phosphatidylinositol.

We first defined growth conditions in which liquid cultures showed robust phase-separated vacuoles, as imaged by the well-established Ld marker Pho8-GFP (Fig. 1*A*). Cells in early stationary stage, after 24 h of growth at 30 °C in minimal medium, showed robust vacuole phase separation but still had cell walls that were readily digestible, unlike those incubated for longer time periods or grown under more complete media. Cells in late exponential stage, after 17 h of growth, contained vacuoles of similar size to stationary stage cells, which is ideal for utilizing the same density centrifugation protocol, but had no detectable domains. We then optimized a set of high and low speed density centrifugation steps (Fig. 1*B*) to generate homogenous vacuole populations that were free of other organelles and LDs. A key addition was a final low speed centrifugation step after the two high speed ones in order to remove bound LDs. Initial assaying of purity was done by microscopy and then validated by western blotting against vacuole (Vac, Pho8) and non-vacuole (mitochondria (Mito), Cox4; ER, Dpm1; Golgi, Gos1-RFP; plasma membrane (PM), Pma1; LD, Erg6-RFP) proteins (Figs. 1*C* and S1). Purified vacuoles for early stationary stage cells retain micron-scale domains that exclude Pho8-GFP (Fig. S2), though domain morphology is lost upon cell dissociation as previously observed (16).

We next carried out a comprehensive lipidomic analysis of polar and non-polar lipids from purified exponential and stationary stage vacuoles (Fig. 1*D*), as well as corresponding whole cell samples (Fig. 1*E*). Distinct features of the vacuole lipidome, such as the low abundance of phosphatidic acid (PA), were consistent between exponential stage vacuoles in our data set, those generated by MemPrep (27, 28), and earlier analyses that were also performed by density centrifugation (29). Exponential and stationary stage vacuoles isolated by our protocol contained low amounts (<0.1%) of cardiolipin (CL), a marker of mitochondrial contamination, less than those in MemPrep-isolated vacuoles (27) or in previously published vacuole lipidomes (30). Because vacuoles form contacts with LDs for degradation of storage lipids, including ergosterol esters (EE) and triacylglycerides (TAG) (31), LD contamination is especially challenging for vacuole isolation. Purified vacuoles showed lower levels of neutral lipids—0.6 mol% EE and 1.2 mol% TAG in exponential vacuoles and 2.3 mol% EE and 5.5 mol% TAG in stationary vacuoles—than previously reported.

### Phase-separated vacuoles are characterized by an enrichment of raft-forming lipids

**Figure 2:**
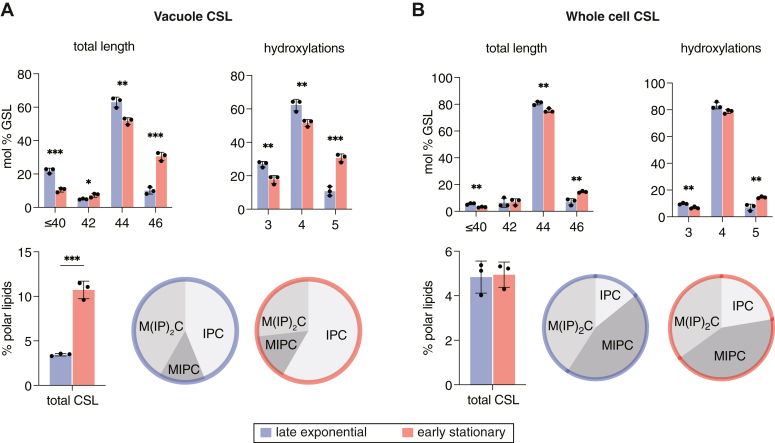
**Changes to SL composition from late exponential to early stationary stage**. *A*, analysis of the CSL pool from late exponential to early stationary stage vacuoles, including lengthening of total chain length, increase in the number of hydroxylations, and changes in relative headgroup composition displayed as a pie-chart. Error bars indicated SD for n = 3 replicate preparations from individually grown cultures. Significance was assessed by unpaired two-tailed *t* test; ∗*p* < 0.05; ∗∗*p* < 0.01; ∗∗∗*p* < 0.001. ∗∗∗∗*p* < 0.0001. *B*, the corresponding data for the CSL pool in whole cell lipidomes, which shows only minimal changes to abundance, length, hydroxylation, and headgroup stoichiometry from late exponential to early stationary stage cells.

The lipidome of centrifugation-purified stationary stage vacuoles from W303a (WT) showed a substantial increase of putative raft-forming lipids, including both ergosterol and SLs (Fig. 1*D*). Compared to late exponential stage vacuoles, the amount of ergosterol increased by 40% and the amount of CSLs showed a 3-fold increase (3.5 ± 0.1–10.7 ± 1.0 mol% of all polar lipids). While all three of the major CSLs showed significant increases, the largest was in IPC (4.0-fold). Thus, the distribution of the CSL pool shifted from one with equal amounts of IPC and M(IP)_2_C in exponential stage, as previously reported (29), to one dominated by IPC in early stationary stage vacuoles (Fig. 2*A*). In the corresponding whole cell lipidomes, the amount of ergosterol, MIPC, and M(IP)_2_C did not increase from late exponential to early stationary stage (Fig. 1*E*). IPC did show a modest increase, but to a smaller extent (1.6-fold) than in the vacuole, and the overall composition of SL headgroups were similar between the two growth stages (Fig. 1*E*). In the vacuole, the average carbon chain length and number of hydroxylations of CSL chains also increased in stationary stage vacuoles (Fig. 2*A*). These changes were also present in the whole cell lipidome, as previously observed (23), but to a smaller extent (Fig. 2*B*). These results indicate that raft-forming lipids are, as a whole, sorted into the vacuole membrane during stationary stage growth, potentially driving phase separation.

We also observed changes in GPLs of stationary stage vacuoles (Fig. 1*D*), though these were more modest than those for sterols and SLs. Phosphatidylethanolamine (PE) decreased in stationary stage vacuoles, which was also observed in MemPrep-isolated vacuoles (28), while it slightly increased in the whole cell. However, we did not observe a corresponding increase in phosphatidylserine (PS) or the substantial increase in PC that was previously reported in late stationary phase vacuoles. As discussed further below, these discrepancies could result from the growth stage analyzed, since they are also reflected in the whole cell lipidome data (Fig. 1*E*). We could also observe changes to the acyl chains of major vacuole GPLs as yeast enter stationary stage (Fig. S3), which were also observed in whole cell lipidomes (Fig. S4). Overall, the PL composition of late exponential stage vacuoles in our growth conditions were similar to those reported in earlier studies (29).

### Genetic modulation of CSL composition in the yeast lipidome

**Figure 3:**
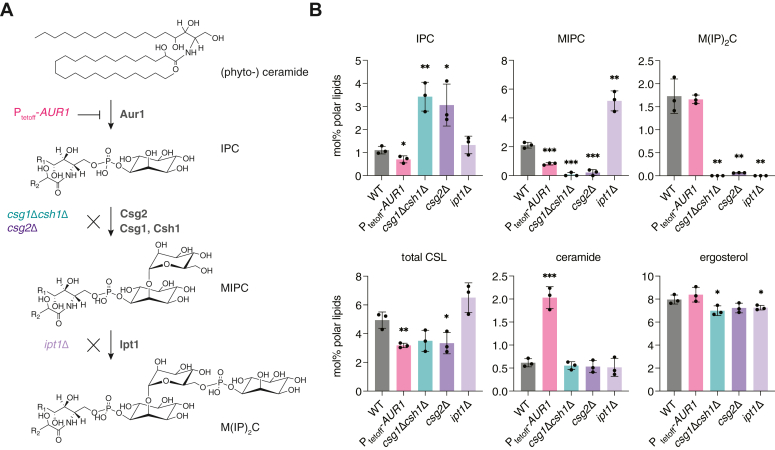
**Genetic dissection of SL composition in a set of yeast strains.***A*, the biosynthetic pathway from phytoceramide to the CSLs IPC, MIPC, and M(IP)_2_C. Successive action by the Aur1, the Csg2 with Csg1 or Csh1 complex, and Ipt1 produce the three abundant CSLs. To the left of each step are shown the strain modifications that manipulate them: knockdown of *AUR1* (P_tetoff_-*AUR1* grown in the presence of doxycycline), deletion of *CSG2* (*csg2*Δ) or *CSG1* and *CSH1* (*csg1*Δ*csh1*Δ), and deletion of *IPT1* (*ipt1*Δ). *B*, whole-cell lipidomics of the strains indicated in panel A, showing the expected changes in the abundance of each SL, as well as total CSL and ergosterol abundances. Significance was assessed by unpaired two-tailed *t* test against WT; ∗*p* < 0.05; ∗∗*p* < 0.01; ∗∗∗*p* < 0.001.

To directly test the hypothesis that changes in SL composition can drive vacuole phase separation, we generated a set of yeast strains to systematically modulate the abundance of each CSL in cells (Fig. 3*A*). The first phosphotransferase in the pathway, Aur1, transfers an inositol phosphate from PI to ceramide phytoceramide, generating IPC (32). *AUR1* is an essential gene, so we utilized a promoter replacement strategy to place its expression under control of the repressor doxycycline (P_tetoff_-*AUR1*). The subsequent glycosyltransferase and phosphotransferase to generate MIPC and M(IP)_2_C, respectively, are non-essential and so gene knockouts could be utilized to test the function of these species. Csg1, Csh1 and Csg2 are the subunits that form MIPC synthase (33); the former two are paralogs that each form complexes with the latter. To create strains that lack MIPC synthesis, we generated mutants lacking Csg1 and Csh1 (*csg1*Δ*csh1*Δ) or in Csg2 (*csg2*Δ). Synthesis of M(IP)_2_C occurs by a single gene product, Ipt1 (34), so loss of M(IP)_2_C was assayed in *ipt1Δ*. The strains generated to analyze CSL effects on vacuole domain formation are summarized in Table S1.

To verify that mutations altered the pathway, we first analyzed the lipidomes of whole cells grown under domain-formation conditions (Fig. 3*B*). In general, lipid abundances changed as expected: P_tetoff_-*AUR1* cells grown in the presence of doxycycline (*AUR1* knockdown) for 24 h showed a substantial (1.5-fold) decrease in total CSLs compared to wild-type (WT) cells and a corresponding increase in ceramides, the substrates for Aur1. Interestingly, the CSL pool in *AUR1* knockdown cells was dominated by M(IP)_2_C. Both *csg2*Δ and *csg1*Δ*csh1*Δ strains had very similar lipidomes containing low abundances amounts of MIPC and only trace amounts of M(IP)_2_C, with a corresponding increase in IPC compared to WT. As previously reported, *ipt1*Δ cells did not produce M(IP)_2_C and accumulated the precursor MIPC, with no changes to IPC levels. Subtle changes to phospholipid species were observed in response to CSL manipulation, suggesting compensatory changes in lipid metabolism (Fig. S5). Most notably, PI levels increased in P_tetoff_-*AUR1*, *csg2*Δ, or *csg1*Δ*csh1*Δ strains compared to WT at the expense of either PE (*AUR1* knockdown) or PC (*csg2*Δ, or *csg1*Δ*csh1*Δ). Because PI is a substrate for Aur1, its increase upon *AUR1* knockdown was expected, but the similarity of that effect to those in *csg2*Δ or *csg1*Δ*csh1*Δ, which do not accumulate IPC, suggests additional interactions between PI and yeast SL metabolism. Ergosterol levels showed only minor changes in these mutants.

### Vacuole CSL composition correlates with domain frequency and morphology

**Figure 4:**
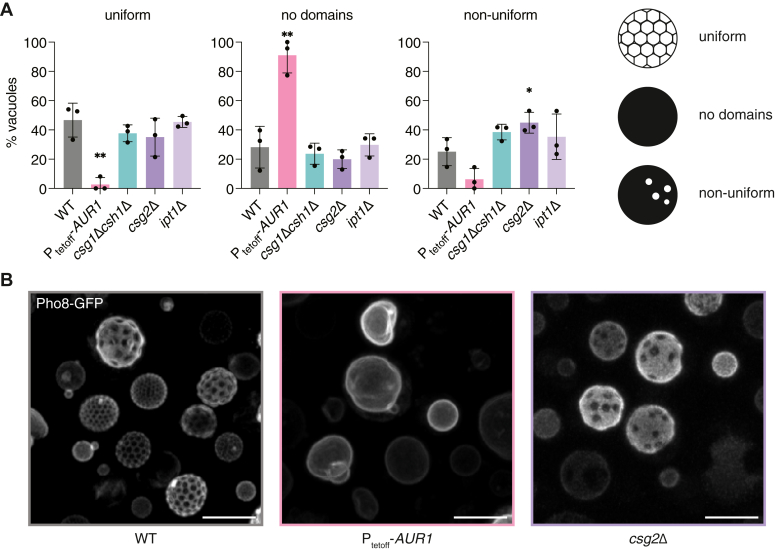
**Yeast SL mutants show changes to vacuole domain abundance and morphology**. *A*, resulting changes in vacuole domain frequency and type for each mutant. Examples of each domain type are shown in Fig. S6. Domain morphology classifications were quantified and plotted for the strains (n = 3 individual cultures, N > 100 cells for each). Significance was assessed by unpaired two-tailed *t* test against the WT; ∗*p* < 0.05; ∗∗*p* < 0.01. *B*, a representative field view of WT, P_tetoff_-*AUR1* grown with doxycycline (*AUR1* knockdown) and *csg2*Δ cells, shown as 3D projections of confocal Z-stacks that were visualized using Ld vacuole domain marker, Pho8-GFP. Scale bars, 5 μm.

We next characterized the effects of CSL perturbation on the ability of cells to undergo vacuole phase separation (Fig. 4*A*). We classified cells as showing (1) uniform Lo domains, forming a distinctive soccer ball-like pattern, (2) non-uniform domains, which were generally smaller, irregularly spaced, and often aggregated, or (3) no domains, characterized by an apparent homogeneous distribution of Pho8-GFP (Fig. S6). Quantification of large numbers of vacuoles from each strain (N > 100 per replicate) showed that WT and *ipt1*Δ cells had similar vacuole morphologies, in which the majority of the vacuoles contained uniform domains. In contrast, *csg2*Δ and *csg1*Δ*csh1*Δ cells, which largely lack MIPC and M(IP)_2_C, exhibited more vacuoles with non-uniform domains. Cells with reduced *AUR1* expression contained vacuoles that predominantly lacked domains. These results indicate that alteration of CSL metabolism is sufficient to modulate the morphology and frequency of vacuole domains.

**Figure 5:**
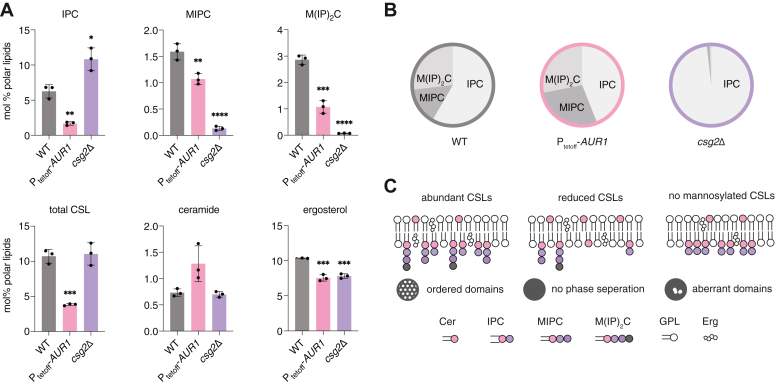
**Correlating vacuole lipid composition with propensity for domain formation in SL mutants**. *A*, lipidomics of isolated vacuoles show changes to SLs and ergosterol levels in vacuoles purified from *AUR1* knockdown and *csg2*Δ cells. The total CSL abundance is significantly reduced in *AUR1* knockdown vacuoles, whereas no change was observed in *csg2*Δ compared to the WT. Significance was assessed by unpaired two-tailed *t* test against the WT; ∗*p* < 0.05; ∗∗*p* < 0.01; ∗∗∗*p* < 0.001; ∗∗∗∗*p* < 0.0001. *B*, in addition to the total amount, the distribution of SL headgroups changes in vacuoles isolated from these mutants, with the proportion of IPC mildly reduced and dramatically increased in *AUR1* knockdown and *csg2*Δ vacuoles, respectively. *C*, proposed model for how SL composition in the vacuole membrane alters domain organization.

Based on the results above, we isolated stationary stage vacuoles from a subset of our mutants to directly correlate changes in domain formation with the membrane lipidome. We compared early stationary stage vacuoles from WT cells with those from *AUR1* knockdown cells, featuring reduced domain frequency, and from *csg2*Δ cells, featuring altered domain morphology (Fig. 4*B*). *AUR1* knockdown vacuoles were characterized by a large (3-fold) reduction in total CSL content compared to WT vacuoles. Of the CSLs, IPC showed the largest reduction, but significant decreases were present in all species. In contrast, *csg2*Δ vacuoles showed an identical total CSL content as in WT, but with an accumulation of IPC and almost complete loss of MIPC and M(IP)_2_C. Thus, both the abundances and distributions of CSLs in these vacuoles differed dramatically from each other (Fig. 5, *A* and *B*). The vacuole lipidomes of SL mutants contained common signatures in other lipid classes when compared to WT vacuoles (Fig. S7): moderately lower ergosterol content (25% less than WT), an increase in the PC/PE ratio, and an increase in PS. The *AUR1* knockdown vacuoles also showed an increase in PI, a substrate for IPC synthase.

While the complexities of real cell membrane composition make direct comparisons of individual lipids challenging, the similar levels of ergosterol and PC/PE ratios of *AUR1* knockdown and *csg2*Δ vacuoles argue that CSL abundance (3-fold higher in the latter, Fig. 5*A*) directly contributes to the frequency of vacuole phase separation (4-fold higher in the latter). Similarly, the comparison between WT and *csg2*Δ vacuoles, which have identical levels of CSLs but different stoichiometries between them, suggests that head group chemistry controls the abundance of non-uniform membrane domains (2-fold higher frequency of cells with this phenotype in *csg2*Δ). Compared to the uniform domains observed in WT cells, the non-uniform *csg2*Δ domains generally occupied a lower surface area, were often irregularly shaped, and showed a low propensity to coarsen upon fusion with other domains they came in contact with (Fig. S8), indicating that they lack fluidity associated with Lo domains in WT vacuoles. These data suggest that abundant CSLs promote membrane domains and a balance between IPC and mannosylated species is required to for proper domain structure and/or dynamics (Fig. 5*C*).

### Abundant CSLs are required for vacuole-associated micro-lipophagy

**Figure 6:**
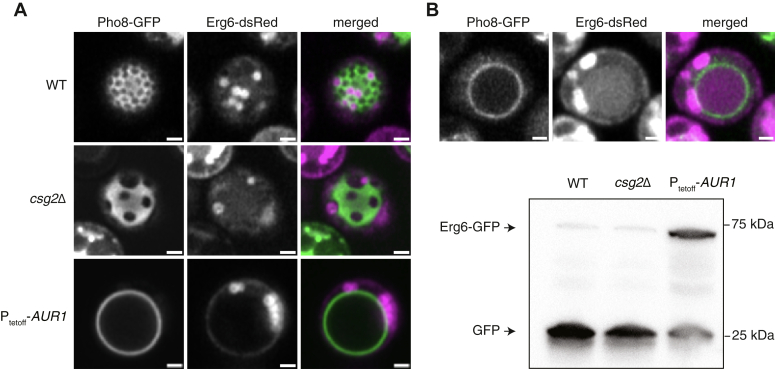
**LD docking and micro-lipophagy is impaired by altered CSL metabolism**. *A*, LDs associate with both uniform and non-uniform Lo domains in WT and *csg2*Δ, cells, but fail to dock in P_tetoff_-*AUR1* cells grown with doxycycline (*AUR1* knockdown), which lack Lo domains. Scale bars, 1 μm. *B*, once LDs are docked and internalized, they are digested inside the vacuole. Shown above is a WT cell after 24 h glucose restriction; the vacuole lumen contains degraded LDs, as indicated by the free dsRed signal inside of the vacuole. In the western blot below, intact LDs are measured in bulk by abundance full length Erg6-GFP, while free GFP is indicative of digestion of LDs and degradation of Erg6-GFP. Compared to WT and *csg2*Δ, more intact Erg6-GFP remains in *AUR1* knockdown cells. Scale bars, 1 μm.

Vacuole domains are required for long-term survival under glucose restriction (17, 21), so we asked if there were differences between microautophagy-related phenotypes between *AUR1* knockdown, *csg2*Δ, and WT cells. We observed that LDs, labeled with Erg6-dsRed, dock to uniform vacuole domains, which are dominant in WT cells, but also to non-uniform domains, common in *csg2*Δ cells (Fig. 6*A*). In contrast, cells lacking vacuole domains, which are dominant in cells with reduced *AUR1* expression, featured LDs that remained peripheral to the vacuole, but never fully docked to its membrane. We assayed micro-lipophagy during starvation conditions by measuring the partial degradation of LD-associated Erg6-GFP into free GFP, which occurs by vacuole proteases upon LD internalization (Fig. 6*B*). In comparison to WT, *AUR1* knockdown cells showed low accumulation of free GFP. In contrast, *csg2*Δ cells showed no defects in LD degradation by this assay. These observations suggest that while vacuole domains, reduced in *AUR1* knockdown cells, are necessary for LD binding and internalization, their characteristic morphology, lost in *csg2*Δ cells, is not.

## Discussion

An emerging model for vacuole organization is that autophagic lipid flux not only requires ordered membrane domains, which act as docking sites for LDs and other cargoes, but also promotes their formation through subsequent incorporation of raft-promoting lipids to the vacuole membrane (19, 35). Here we provide the first detailed lipidomics data supporting this hypothesis, finding that both ergosterol and SLs re-distribute to the vacuole membrane at the onset of the early stationary stage. Key to these data was the identification of growth conditions in which robust domain formation can be observed while the cell wall is still readily digestible for organelle purification. We compared vacuoles from these conditions to those isolated from late exponential stage cells, where vacuoles are of similar size and density but completely lack domains. We found that the switch to early stationary stage corresponds to an increase in raft-forming components, including ergosterol and all three yeast CSLs, a potential trigger for membrane phase separation. Notably, these components did not increase in the corresponding whole cell lipidome, indicating that they are re-directed into the vacuole. The abundances of each lipid species in the samples analyzed here are provided in Data File S1.

Our study is the second recent attempt to characterize the lipidome of stationary stage vacuoles. The other (28) used a very different isolation technique that relies on immunoprecipitation of vacuole-derived vesicles; it did not show any increase in ergosterol content and only modest changes to the CSLs of stationary stage vacuoles. In addition to methodological differences, a clear source of variability between the studies is the difference in growth conditions. Here, phase separated vacuoles were isolated from early stationary stage cells, harvested after only 24 h of growth on low glucose minimal media. In contrast, immunoprecipitated vacuole membranes were isolated from late stationary stage cells after 48 h of growth in richer, complete media containing higher levels of glucose and amino acids. The yeast lipidome changes dramatically during the stationary stage between 24 and 48 h, a difference that is likely even larger given the differences in growth medium. Notably, an increase in high melting temperature PC lipids observed in immunoprecipitated vacuole membranes vacuoles, but not in our early stationary stage vacuoles, have also been observed in whole cells sampled at 48 h of growth, but not at 24 h (23). While vacuole phase separation could be achieved through different mechanisms depending on the growth stage, its rapid onset over the course of just a few hours likely necessitates intracellular sorting of lipids from existing pools, which our data supports.

We were surprised by the magnitude by which CSLs become enriched in early stationary stage vacuole membranes compared to domain-free vacuoles harvested only hours previously. The composition of the CSL pool also changed, shifting to one enriched in IPC with longer and more hydroxylated chains. As for any analysis based on organelle purification, it is challenging to confirm homogenous sampling across cells and rule out all potential impurities. Therefore, we sought to directly test if CSLs act as modulators of membrane phase separation. We found that the reduction of IPC and total CSL content in the vacuole, achieved through reduced expression of *AUR1*, strongly inhibited domain formation and its associated phenotypes of LD micro-lipophagy. In contrast, an increase in vacuole IPC content, which is found in *csg2*Δ cells, did not change domain abundance but instead altered their appearance and dynamics. Due to their fluid nature, Lo domains are highly mobile in the vacuole, and upon collision, readily merge in order to minimize the line tension at the phase boundaries (36). The irregular domains abundant in *csg2*Δ cells also diffuse on the vacuole membrane but, upon collision, do not rapidly coalesce. Instead, they retain their initial shapes (Fig. S8), suggesting that they could represent more solid gel-like domains. Previous studies have suggested that gel-like domains are especially promoted by IPC: the high Laurdan General Polarization values of IPC/ergosterol mixtures (37) and direct observations of gel-like nanodomains on the yeast PM that are inhibited by mannosylation of IPC into MIPC and M(IP)_2_C (38). Gel-like domains are also found to be predominant in ternary mixtures of GPLs, cholesterol, and glucosylceramide, the closest analogue to IPC in metazoans (39). Although capable of importing LDs through micro-lipophagy, these aberrant domains could impede membrane re-mixing upon reintroduction of nutrients.

All three CSLs (IPC, MIPC, and M(IP)_2_C) are among the most abundant lipids in *S. cerevisiae* cells, but it is not yet known how their composition is regulated in different growth stages, nor how they are sorted into specific organelles like the vacuole. The loss of vacuole domain formation in mutants of both MVB (14) and autophagy pathways (17), both of which direct membrane cargoes to the vacuole under starvation conditions (40), suggests potential mechanisms for CSL sorting that can be tested via lipidomics of purified vacuoles. The biophysical properties of yeast CSLs have also not yet been extensively investigated, partially as a result of the lack of available synthetic versions for use in model membrane experiments. In experiments utilizing yeast-derived lipid mixtures, it has been observed that IPC specifically increases membrane ordering in liposomes, especially those containing ergosterol (37). It has also been shown that the ability of yeast lipid extracts to phase separate when reconstituted in GUVs is lost in *elo3*Δ cells that synthesize CSLs with shorter chains (22, 37). Thus, multiple lines of data support the role of this lipid class in the formation of ordered membrane domains. An open question is what biophysical constraints dictate the stoichiometry between the three yeast CSL classes, each of which features pronounced differences in head-group size, hydrogen-bonding capacity, and anionic charge.

Yeast CSLs bear structural similarities to glycosylated lipids in the other eukaryotic lineages, all of which have been implicated in inducing ordered membrane phases and potential domains. Glycosyl inositol phosphorylceramides, the most abundant sphingolipids in plant cells, have been shown to promote membrane ordering in model membranes (41, 42) and can cluster in nanodomains on the plant PM (43). Mammalian glycosylated sphingolipids, gangliosides, are also potent nucleator membrane domains. Despite their different biochemistry and increased complexity, gangliosides share some common structural features with yeast CSLs, including variable combinations of both hexose subunits and charged groups (sialic acids or phosphates). Extensive experimental and modeling studies of gangliosides (44), and their hexosyl ceramide precursors (39), have highlighted the complex roles their headgroup composition can have on local membrane structure and formation of membrane domains *in vitro*. Understanding the “sugar-code” by which diverse glycosylated lipids can influence membrane organization could be aided by similar approaches applied to fungal CSLs, as yeast feature a tractable compartment for investigating their structural effects on membrane organization.

## Experimental procedures

### Yeast strains, plasmids, and media

*S. cerevisiae* W303a was used as the base strain throughout the study. All strains generated are listed in Table S1. Integrations were generated by homologous recombination of PCR-amplified cassettes transformed via the lithium acetate method. The gene of interest was replaced by selection markers to generate knockout strains. For P_tetoff_-*AUR1* strain, a tetO_2_-CYC1 promoter and expression cassette for the tetracycline-controlled transactivator were amplified from plasmid PCM224 was substituted into the 500 bp upstream from the *AUR1* start codon. For vacuole imaging, yeast strains were transformed with plasmid pRS426 GFP-Pho8 that allows expression of Pho8-GFP. YPD, complete synthetic medium (CSM), and minimal medium were used to grow yeast cells. YPD medium (Fisher Scientific) contained yeast extract (10 g/L), peptone (20 g/L) and glucose (20 g/L). CSM medium contained glucose (20 g/L), ammonium sulfate (5 g/L) and yeast nitrogen base (Dibco) (1.7 g/L). For CSM medium, amino acids and nucleobases were supplemented using CSM powders (MP Biomedicals). In order to promote vacuole phase separation, yeast were diluted into minimal medium that contains 0.4% glucose, ammonium sulfate, yeast nitrogen base and only essential amino/nucleic acids. Minimal medium contained glucose (Fisher Scientific) (4 g/L), ammonium sulfate (Fisher Scientific) (5 g/L), yeast nitrogen base without ammonium sulfate and amino acids (BD Difco) (1.7 g/L), leucine (Alfa Aesar) (20 mg/L), histidine (Acros Organics) (20 mg/L), tryptophan (Alfa Aesar) (20 mg/L), adenine (Alfa Aesar) (10 mg/L) and uracil (Alfa Aesar) (20 mg/L), as described by Sherman *et al*. (45) Inhibition of *AUR1* expression was induced by addition of 10 mg/ml of doxycycline (Sigma-Aldrich) stock solution to a final concentration of 10 μg/ml when the culture was diluted into minimal medium. Doxycycline added to WT cells at this concentration showed no effects on vacuole morphology or phase separation.

### Vacuole purification by density centrifugation

Yeast strains expressing Pho8-GFP were used to confirm the growth stage and domain formation under confocal microscopy before starting vacuole purification. An overnight culture in 5 ml YPD was prepared, grown in a shaker for ∼18 h, then diluted 1/100 into 42 ml CSM - uracil in a 125 ml flask. After ∼18 h incubation, 13 OD units of yeast were diluted to 650 ml minimal media in a 2 L non-baffled flask shaking at 200 rpm. For yeast in the late exponential phase, the cultures were incubated for 17 h after dilution into minimal medium (OD ∼ 0.7). For early stationary phase vacuole samples, yeasts were harvested after 24 h of incubation (OD ∼ 1.2). The cells were harvested by benchtop centrifugation (3,000*g* for 30 min), yielding 2-3g of wet weight (wwt) cell pellet.

Initial vacuole purification was done by a method expanded from Wiederhold *et al*. (46) with several modifications. The cell pellet was first resuspended in 10 ml of 100 mM Tris-HCl, pH 9.5, then 100 μL of 1M DTT (Fisher Bioreagents) was added. After incubation at 30 °C for 10 min, cells were centrifuged at 4,000×*g* for 5 min, washed with 10 ml of water, washed with 10 ml of 1.1 M sorbitol (Thermo Scientific), then resuspended in 5 ml of 1.1 M sorbitol. To generate spheroplasts, 9 mg Zymolyase 20T (Amsbio) per gram wwt cells was dissolved in 1 ml of 1.1 M sorbitol, then added to the suspension. When purifying vacuoles from exponential phase yeast, 5 mg Zymolyase 20T per gram wwt cells was used instead. After 1 h of incubation at 30 °C (with manual swirling every 15 min), all procedures were conducted on ice or at 4 °C. Spheroplasts were washed by centrifuging the spheroplasts through a layer of 7 ml of 7.5% Ficoll 400 (VWR) in 1.1 M sorbitol at 4,000×*g* for 20 min. The spheroplasts were resuspended in 10 ml 12% Ficoll 400 in 10 mM Tris-MES, pH 6.9. For lysis, the suspension was transferred to a dounce homogenizer (15 ml, Wheaton) and homogenized by 15 strokes using a tight (“B”) pestle. 10.7 ml suspension was transferred to a centrifuge tube (Beckman Coulter) with 5.3 ml of 12% Ficoll 400 in 10 mM Tris-MES was layered on top of it. Centrifugation was performed at 50,000×*g* for 35 min in a SW 32.1Ti rotor (Beckman Coulter). Crude vacuoles were isolated from the top layer and collected using a pipet (∼1 ml) and diluted in 5 ml 12% Ficoll 400 in 10 mM Tris-MES.

Crude vacuoles were further purified to generate pure samples, free of other contaminants, especially associated LDs. Crude vacuoles were first lightly homogenized in a dounce homogenizer by stroking 5 times with the B pestle. 5.3 ml of vacuole suspension was transferred to a centrifuge tube, then layered with 5.3 ml 8% Ficoll 400 in 10 mM Tris-MES and 5.3 ml 4% Ficoll 400 in 10 mM Tris-MES. The vacuoles were further separated from the mixture through density centrifugation at 50,000×*g* for 35 min in a SW 32.1Ti rotor. After centrifugation, the top layer was collected. In order to further purify the vacuole fraction, we adapted a low speed centrifugation protocol by Wiemken *et al*. (47). For this, vacuoles were diluted with 0.6 M sorbitol in 5 mM PIPES-AMPD, pH 6.8. They were then purified through sorbitol/sucrose gradient: two volumes (2–5 ml) of the diluted vacuoles were layered on top of one volume of 0.4 M sorbitol, 0.2 M sucrose (Thermo Scientific) in 5 mM PIPES-AMPD, pH 6.8, which was layered on top of one volume of 0.36 M sorbitol, 0.24 M sucrose in 5 mM PIPES-AMPD, pH 6.8 (47). After centrifugation at 3500×*g* for 30 min, vacuoles sedimented at the bottom of the tube. The vacuoles were resuspended in 0.6 M sorbitol buffer. Vacuole integrity was assessed immediately by imaging under wide-field fluorescence and transmitted light microscopy (Thermo EVOS equipped with 63× Nikon oil immersion objective) after each purification. Successful preparations showed predominantly large particles (vacuoles) that were Pho8-GFP positive. These were then snap frozen and kept at −80 °C before analysis.

### Western blot analysis of vacuole purity and LD micro-lipophagy

The purity of vacuoles was assessed by means of western blot analysis. 20 μg protein, as measured by BCA assay, was loaded for each sample. Primary antibodies against different organelle markers included anti-Cox4 (Abcam, ab110272), anti-Dpm1 (Abcam, ab113686), anti-Pma1 (Invitrogen, MA1-91567) and anti-Pho8 (Abcam, ab113688). Golgi and LD contaminations were assessed by a primary antibody against RFP (Rockland, 600–401–379) in strains harboring a Gos1-RFP or Erg6-RFP plasmid. For LD microautophagy assay, 2.5 OD units of cells were collected, then proteins were extracted as described previously (48). 10 μl of the protein extract was loaded for each blot and anti-GFP (Invitrogen, GF28R) was used as the primary antibody. As a secondary antibody, goat anti-mouse IgG (H + L), HRP conjugate (Invitrogen, 31,430) or goat anti-Rabbit IgG (H&L) HRP conjugate (ImmunoReagents, Inc., GtxRb-003-EHRPX) were used. Specificity of commerical antibodies were validated by the manufacturer. Blots were developed using the Pierce ECL Western Blottting Substrate (Thermo, 32,106) and imaged on ChemiDoc XRS+ (Bio-rad). Band intensity was quantified in ImageJ.

### Lipid extraction and lipidomics of yeast cells and isolated vacuoles

Mass spectrometry-based lipid analysis was performed by Lipotype GmbH as described (22, 23). Lipids were extracted using a two-step chloroform/methanol procedure (22). Samples were spiked with internal lipid standard mixture containing: CDP-DAG 17:0/18:1, CL 14:0/14:0/14:0/14:0, Cer 18:1;2/17:0, DAG 17:0/17:0, LPA 17:0, LPC 12:0, LPE 17:1, LPI 17:1, LPS 17:1, PA 17:0/14:1, PC 17:0/14:1, PE 17:0/14:1, PG 17:0/14:1, PI 17:0/14:1, PS 17:0/14:1, EE 13:0, TAG 17:0/17:0/17:0, stigmastatrienol, IPC 44:0;2 MIPC 44:0;2 MIPC and M(IP)_2_C 44:0;2. After extraction, the organic phase was transferred to an infusion plate and dried in a speed vacuum concentrator. First step dry extract was re-suspended in 7.5 mM ammonium acetate in chloroform/methanol/propanol (1:2:4, V:V:V) and second step dry extract in 33% ethanol solution of methylamine in chloroform/methanol (0.003:5:1; V:V:V). All liquid handling steps were performed using Hamilton Robotics STARlet robotic platform with the Anti Droplet Control feature for organic solvents pipetting.

Lipid extracts were analyzed by direct infusion on a QExactive mass spectrometer (Thermo Scientific) equipped with a TriVersa NanoMate ion source (Advion Biosciences). Samples were analyzed in both positive and negative ion modes with a resolution of R_m/z=200_ = 280,000 for MS and R_m/z=200_ = 17,500 for MSMS experiments, in a single acquisition. MSMS was triggered by an inclusion list encompassing corresponding MS mass ranges scanned in 1 Da increments (49). Both MS and MSMS data were combined to monitor EE, DAG and TAG ions as ammonium adducts; PC as an acetate adduct; and CL, PA, PE, PG, PI and PS as deprotonated anions. MS only was used to monitor CDP-DAG, LPA, LPE, LPI, LPS, IPC, MIPC, M(IP)_2_C as deprotonated anions; Cer and LPC as acetate adducts and ergosterol as protonated ion of an acetylated derivative (50).

Lipidomics data were analyzed with in-house developed lipid identification software based on LipidXplorer (51, 52). Data post-processing and normalization were performed using an in-house developed data management system. Only lipid identifications with a signal-to-noise ratio >5, and a signal intensity 5-fold higher than in corresponding blank samples were considered for further data analysis. Abundances for each lipid species, expressed as mol %, were compiled and are available in a spreadsheet as part of the Supplementary Information. Subsequent plotting and statistical tests were performed in Graphpad Prism.

### Microscopy of vacuole phase separation

Yeast strains expressing Pho8-GFP, a Ld domain marker (17), were used for fluorescence microscopy. For each biological replicate, an overnight culture was first grown in 5 ml YPD, then diluted 1/100 to 5 ml CSM medium lacking uracil for selection and incubated for ∼18 h 0.1 OD unit of the CSM culture was diluted in 5 ml minimal media and incubated for 24 h prior to imaging. All incubations were done in 30 °C round bottom tubes (Fisher Scientific) shaking at 250 rpm. Prior to imaging, live yeast cells were immobilized in 8-well microscope chamber slides (Nunc Lab-tek, Thermo Fisher Scientific) pre-coated with 1 to 2 mg/ml concanavalin-A (MP Biomedicals). Vacuole images were acquired on a Zeiss LSM 880 confocal microscope with an Airyscan detector. Samples were imaged at room temperature using a Plan-Apochromat 63×/1.4 Oil DIC M27 objective. Excitation was through a 488 nm Argon laser set at 2% power. For each sample, two to four ∼6–8 μm Z-stack images were acquired depending on the cell density, and Airyscan processed in ZEN Black using default settings. For live cell image analysis, cells were categorized into three groups based on their vacuole domain morphology. Only vacuoles captured from top to bottom were used for the quantification, and at least 100 cells were quantified in a given sample. 3D projections were generated using the Z project tool in ImageJ. For purified vacuoles, samples were mounted on a 0.8% low gelling temperature agarose (Sigma, A9045) pad in 5 mM PIPES-AMPD buffer.

### Statistical analysis

All experiments were performed in biological replicates grown from individual yeast colonies. Values are expressed as mean ± standard deviation. Significance analysis was determined by unpaired, two-tailed t-tests using GraphPad Prism 9 software; *p* < 0.05 was considered statistically significant, *p* > 0.05 was considered statistically insignificant.

## Data availability

Lipidomics data for all analyses are provided in Data File S1. Raw data for microscopy and all other analyses are available from the corresponding author.

## Supporting information

This article contains supporting information (16).

## Conflict of interest

The authors declare that they have no conflicts of interest with the contents of this article.

## Supporting information

Data FileS1
