# Supplementary material for: Intracellular sphingolipid sorting drives membrane phase separation in the yeast vacuole
Source: J Biol Chem. 2023 Nov 25;300(1):105496. doi: 10.1016/j.jbc.2023.105496 (PMC10776997; doi:10.1016/j.jbc.2023.105496)
Supplement: Supporting information [file mmc2.pdf]

Supplementary Information

Intracellular sphingolipid sorting drives membrane phase separation in the yeast vacuole

Hyesoo Kim and Itay Budin

This file includes:

Figures S1-S8

Table S1

Description for Data File S1

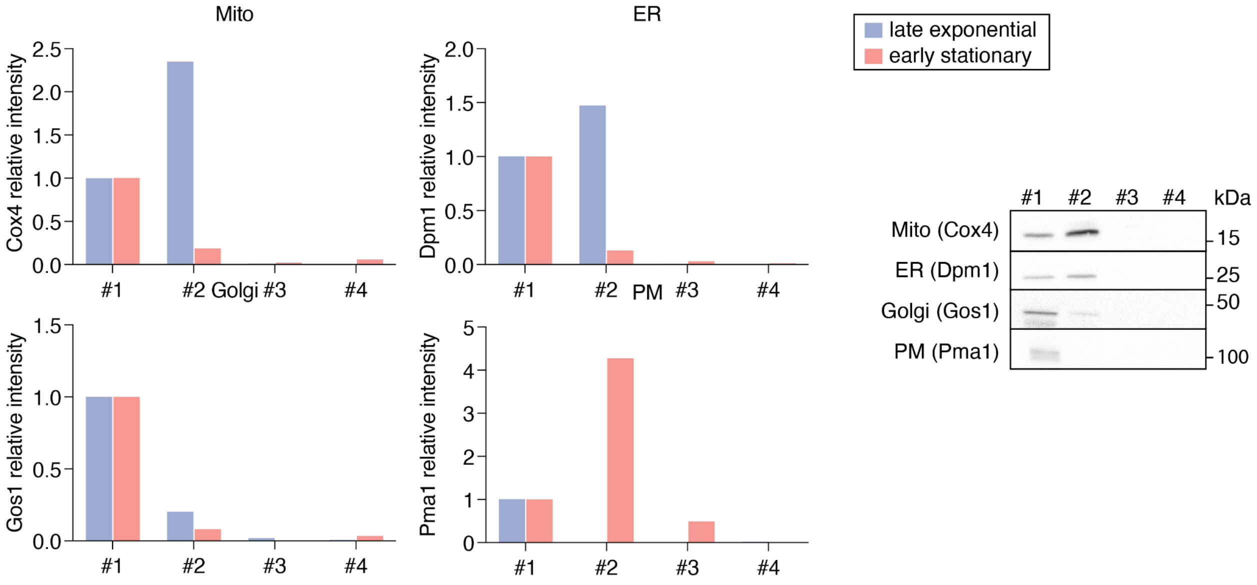

Figure S1: Analysis of organelle impurities in purified vacuoles. Left: Quantification of western blot bands corresponding to mitochondrial, ER, Golgi, and PM proteins in samples fractions during vacuole purifications. Right: western blot of impurities in late exponential stage vacuole purification from W303a.

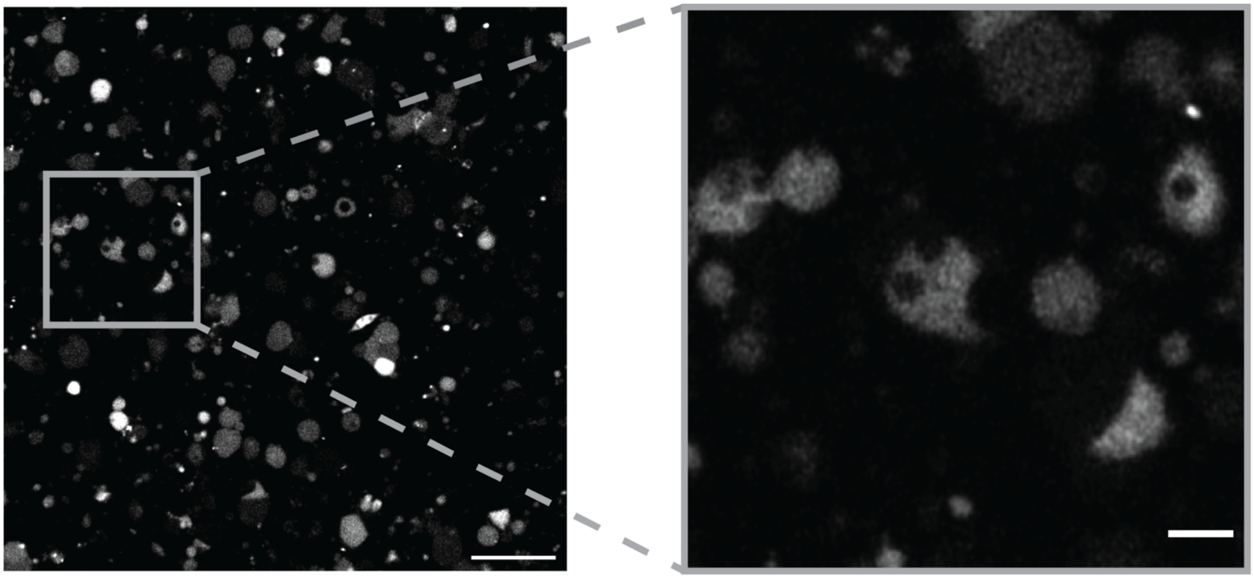

Figure S2: Purified early stationary stage vacuoles retain the ability to phase separate. Shown is an example confocal micrograph from W303a vacuoles from cells expressing Pho8-GFP. Inset shows numerous vacuoles with ordered domains excluding Pho8-GFP or complete phase separated hemispheres. The distinctive polygonal morphology of WT vacuole domains is lost when cells are dissociated, as previously observed (16). Scale bars: whole field, 10 μm; inset, 2 μm.

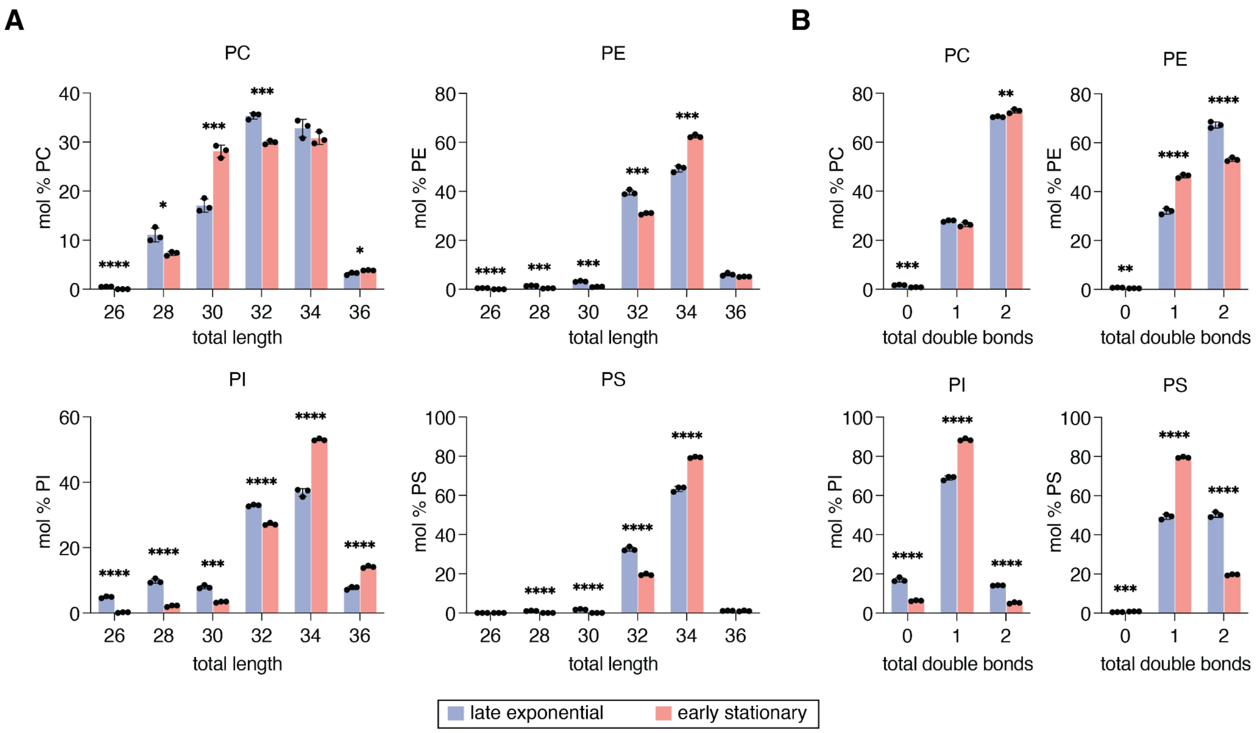

Figure S3: Molecular features of GPLs in isolated vacuoles. (**A**) Total acyl chain length, combining both acyl chains in PC, PE, PI, and PS of late exponential and early stationary stage vacuoles. PA and PG are not shown due to low abundance. (**B**) Number of double bonds (unsaturations) in late exponential and stationary stage vacuoles. The proportion of monounsaturated PE, PI and PS lipids increases in the early stationary stage, accompanied by a large decrease of di-unsaturated species. Significance was assessed by unpaired two-tailed t-test; *, $p < 0.05$; **, $p < 0.01$; ***, $p < 0.001$; ****, $p < 0.0001$.

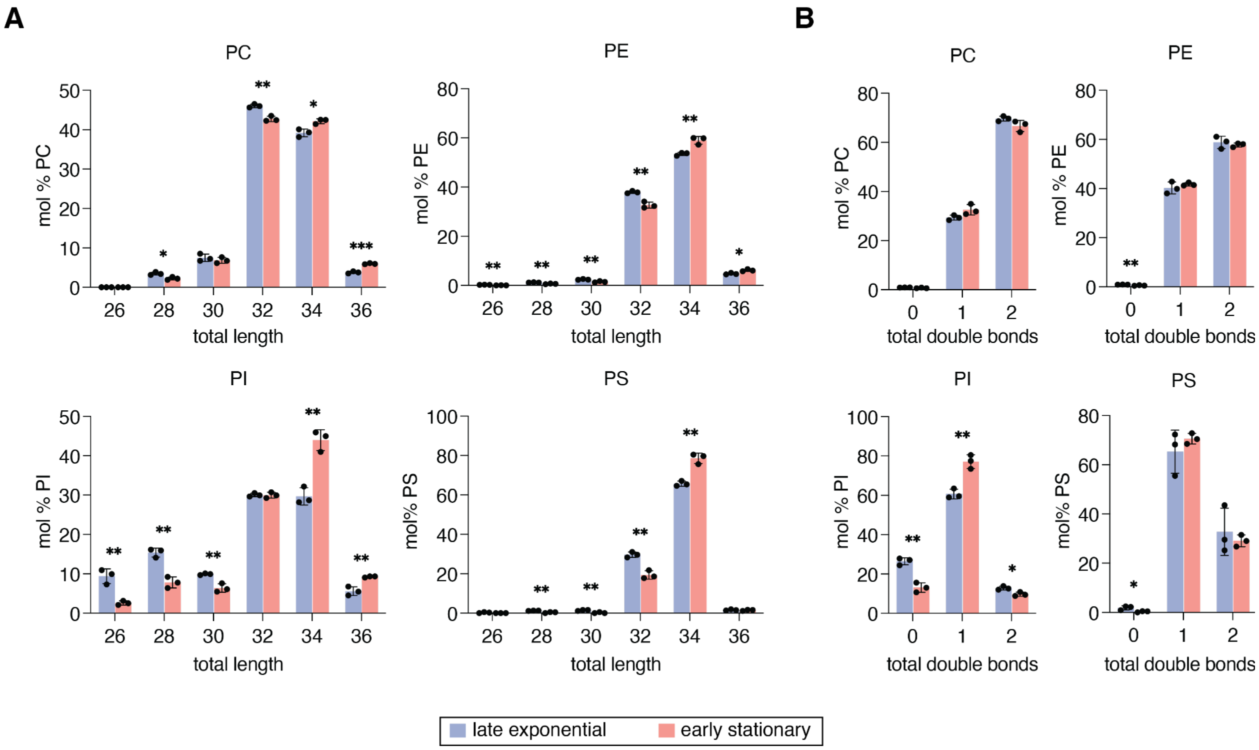

Figure S4: Molecular features of GPLs in whole cell lipid extracts. (**A**) Total acyl chain length, combining both acyl chains in PC, PE, PI, PS of the whole cell. The abundance of GPLs containing longer acyl chains (> 32 C) slightly increases in the stationary phase. (**B**) Total number of double bonds in PC, PE, PI and PS in whole cell lipidome. The unsaturation in PI is affected the most during the growth stage shift from late exponential to early stationary phase. The proportion of PI with only one double bond significantly increases, accompanied by decrease in PI with zero or two double bonds. Significance was assessed by unpaired two-tailed *t* test; *, $p < 0.05$; **, $p < 0.01$; ***, $p < 0.001$.

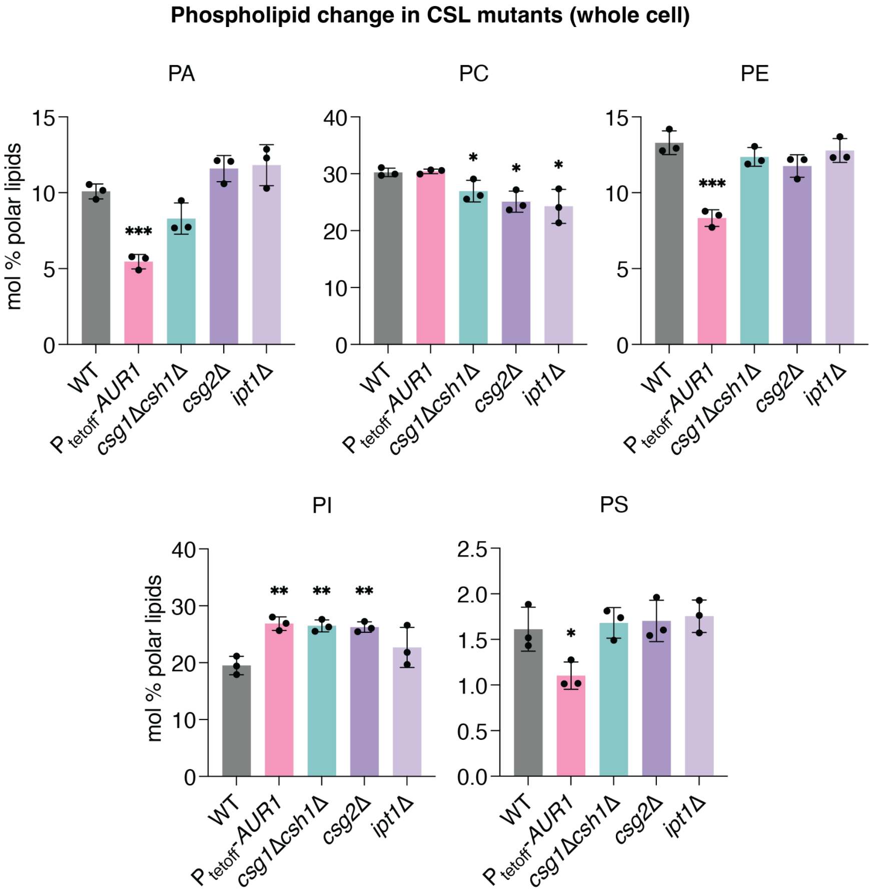

Figure S5: The change in different GPL classes in the whole cell lipidome of CSL mutants. The GPL profile of P_{tetoff}-*AUR1* grown with doxycycline was significantly altered in all GPL classes except PC. The whole cell samples of *csg1*Δ*csh1*Δ and *csg2*Δ showed very similar changes, a decrease in PC and increase in PI compared to WT. Significance was assessed by unpaired two-tailed *t* test against the WT; *, $p < 0.05$; **, $p < 0.01$; ***, $p < 0.001$.

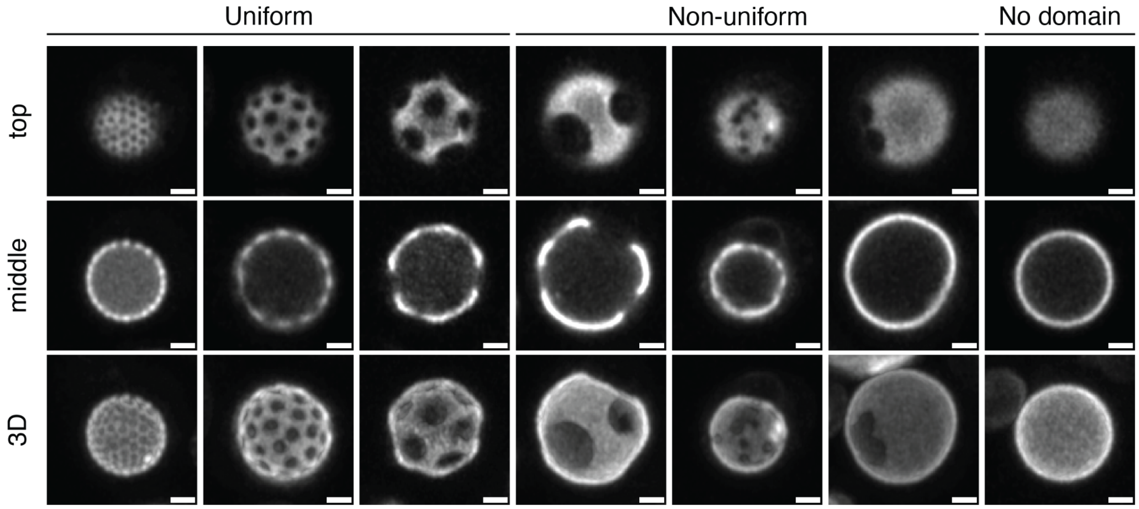

Figure S6: Categorization of vacuoles based on their domain morphology. Examples are shown of vacuole domain morphologies in early stationary stage cells. 'Uniform' vacuoles show domains that are uniformly distributed on the vacuole membrane, 'non-uniform' vacuoles have domains that are irregularly spaced out and 'no domain' vacuoles contain no observable vacuole microdomains under our imaging conditions. Shown are top and middle slices from typical Z-stacks and the resulting 3D projection. Scale bars, 1 μm.

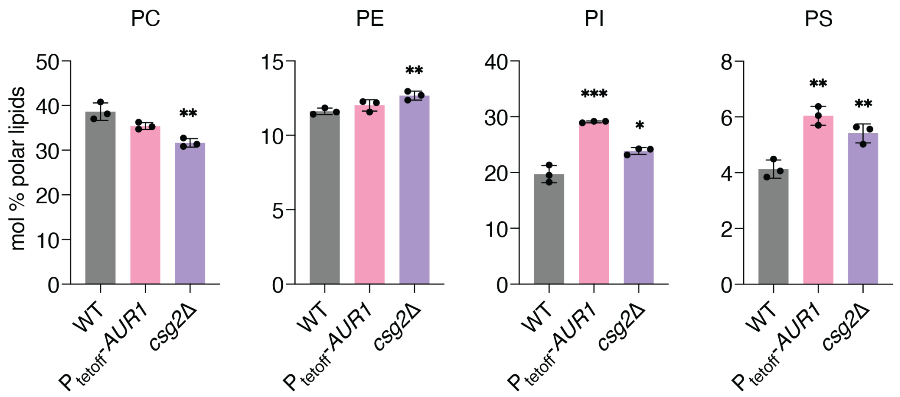

Figure S7: The change in different phospholipids in isolated vacuoles from P_{tetoff}-*AUR1* cells (grown with doxycycline) and *csg2*Δ cells, compared to WT. The change does not follow the trend observed in the whole cell lipidome, except PC and PI. PE increases in *csg2*Δ vacuole, but not the whole cell. PE level is essentially the same in P_{tetoff}-*AUR1*, when it was significantly reduced in the whole cell. The increase in PS was observed in the vacuole, which was not the case in the whole cell lipidome. Significance was assessed by unpaired two-tailed *t* test against the WT; *, $p < 0.05$; **, $p < 0.01$; ***, $p < 0.001$.

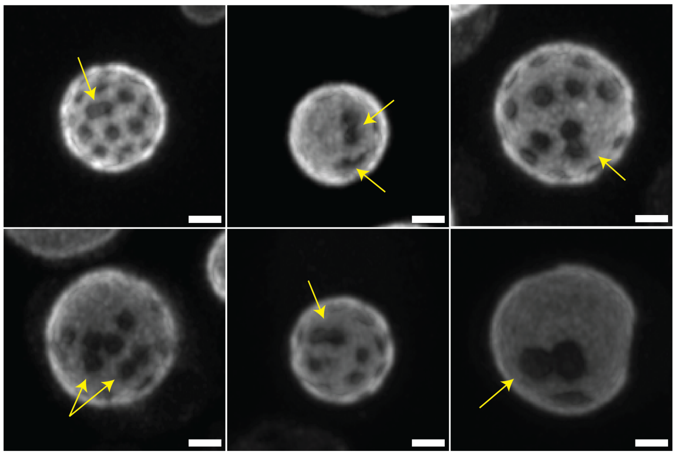

Figure S8: Vacuole domains do not readily coalesce upon collision in *csg2*Δ vacuoles. When liquid Lo domains typically coalesce, they form larger domains that quickly reform to minimize line tension (16). Shown are several examples where *csg2*Δ vacuoles showed ordered domains, imaged using Pho8-GFP (Ld marker), that upon fusion do not show rapid line tension minimization and retain their original shape (arrows). This could be explained by solid gel-like domains in *csg2*Δ vacuoles, in contrast to the liquid Lo domains typically observed in WT vacuoles. Scale bars, 1 μm.

Strain	Genotype
W303a	*MATa ura3-52 trp1Δ2 leu2-3_112 his3-11 ade2-1 can1-100*
P_{tetoff}-*AUR1*	W303a, *aur1::tetO₂-P_{CYC1}-AUR1*
csg2Δ	W303a, *csg2Δ::kanMX4*
csg1Δcsh1Δ	W303a, *csg1Δ::his6MX csh1Δ::LEU2*
ipt1Δ	W303a, *ipt1Δ::kanMX4*
ncr1Δ	W303a, *ncr1Δ::kanMX4*
npc2Δ	W303a, *npc2Δ::his6MX*

Table S1: List of yeast strains used in this study

Data File S1: Spreadsheet containing the abundances of each lipid species, expressed in pmol per sample, in the whole cell and vacuole samples used in this study. For each sample, three biological replicates are provided. From these, mol % of each lipid species were calculated by dividing individual species amounts by the abundances summed for all species in that sample. For calculation of mol % of polar lipids, abundances for TAGs, DAGs, and EEs were excluded.
